# Altered regulatory mechanisms governing cell survival in children affected with clustering of autoimmune disorders

**DOI:** 10.1186/1824-7288-38-42

**Published:** 2012-09-12

**Authors:** Loredana Palamaro, Giuliana Giardino, Francesca Santamaria, Ugo Ramenghi, Umberto Dianzani, Claudio Pignata

**Affiliations:** 1 Department of Pediatrics, “Federico II” University, Pansini 5, 80131, Naples, Italy; 2 Interdisciplinary Research Center of Autoimmune Diseases (IRCAD) and Department of Medical Science, “A. Avogadro” University, Eastern Piedmont, Novara, Italy

**Keywords:** Clustering of autoimmune diseases, Fas, Apoptosis, ALPS

## Abstract

Clustering of Autoimmune Diseases (CAD) is now emerging as a novel clinical entity within monogenic immune defects with a high familial occurrence. Aim of this study is to evaluate the regulatory mechanisms governing cell survival, paying a particular attention to Fas-induced apoptosis, in a cohort of 23 children affected with CAD. In 14 patients, Fas stimulation failed to induce cell apoptosis and in 1 case it was associated with Fas gene mutation. Our study highlights the importance to evaluate cell apoptosis in the group of children with CAD, which, with this regard, represents a distinct clinical entity.

## 

Dear Editor,

Even though distinct autoimmune disorders may be associated in the same individual [1,2], only rare patients exhibit a clear clustering of distinct diseases, which are indicative of a common poly-reactive autoimmune process [3]. Along with environmental factors, a genetic susceptibility represents a well established feature in the predisposition of individuals to certain autoimmune diseases, including the association with certain specific HLA and complement polymorphic variants. However, the intimate pathogenic mechanism of autoimmunity still remains to be unraveled. Alterations of homeostatic mechanism resulting in an abnormal lymphocyte accumulation, autoimmunity or lymphoid malignancies, have now emerged as a novel pathogenic mechanism underlying intense poly-reactive auto-reactions [4-6]. Recent evidence indicates that, in a few cases, Clustering of Autoimmune Disorders (CAD) may represent unique model of monogenic autoimmune disorder or a sign of congenital immunodeficiencies [7-9]. Hematologic autoimmune disorders associated with non-malignant lymph adenopathy are the prominent clinical features of the Autoimmune Lymphoproliferative Syndrome (ALPS), whose molecular characterization leads to define five distinct entities on the basis of the location of the defect in the Fas signaling cascade [3]. However, in a large group of ALPS patients the molecular defect still remains to be identified. We recently reported on a group of children affected with CAD who exhibited a high prevalence of familial cases [10].

Aim of this study is to evaluate Fas-induced apoptosis in this cohort of patients.

CAD was defined by the presence of at least three distinct organ-specific or systemic immune disorders in the same individual [10]. The predominant autoimmune diseases in these 23 patients (14 female) were rheumatoid arthritis, type 1 diabetes, autoimmune thyroiditis and celiac disease, as previously described in detail [10]. Fas-mediated lymphocyte apoptosis was evaluated on activated T-cell lines obtained by treating peripheral blood mononuclear cells (PBMC) with phytohemagglutinin (PHA) at days 0 (1 μg/mL) and 12 (0.1 μg/mL), as previously reported [2,11]. Fas function was defined defective when cell survival was higher than 78%, which was the 95^th^ percentile of the response displayed by normal controls.

Fas-induced cell death in PHA-derived T-cell lines was abnormal in 14 of the 23 patients (60%) (Figure 1). Fas expression was evaluated in the long-term T-cell lines by direct immunofluorescence on the same day in which Fas function was assessed and was expressed always at comparable levels than in controls. In the 14 patients with defective Fas-induced apoptosis, sequencing analysis of the Fas gene (TNFRSF6) revealed a 2 base deletion in exon 4 (g410-411delCT) in one patient (Pt#14). In other patients, 5 already described silent polymorphisms were also found, 2 of them in the 5’ UTR region, 2 in the coding region, and 2 in the intronic region (IVSIII nt 46, IVSV nt 82).

**Figure 1:**
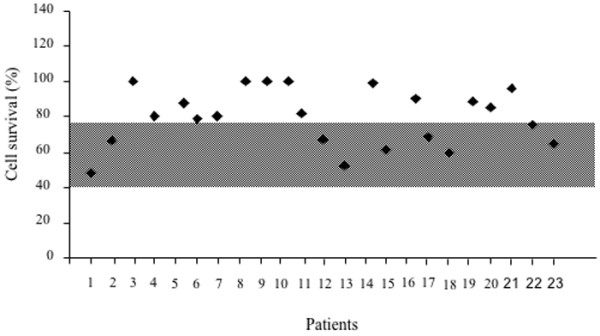
**T-cell sensitivity to Fas induced cell death in CAD patients.** Peripheral blood T cells were stimulated by PHA and cultured in the presence of rIL-2 prior to further stimulation through Fas receptor. Results were expressed as relative cell survival percentage, calculated as follows: (total live cell count in the assay wells/total live cell count in the untreated samples) x 100. Shaded area indicates the range of normal values (i.e. the range between the 5^th^ and the 95^th^ percentile of normal controls values).

In this study we report on a functional impairment of cell death, induced through Fas triggering, in the 60% of patients affected with CAD, thus suggesting some overlap with ALPS [3,4]. Only in 1 patient the functional alteration was associated with gene mutation.

A genetic cause of certain complex autoimmune syndromes has also been established in Autoimmune Polyendocrinopathy-Candidiasis-Ectodermal Dystrophy syndrome (APECED) [12,13]. However, in our patients the diagnostic criteria for this syndrome were missing. Apoptosis is a complex process that plays a central mechanism in the homeostasis of immune response and in the regulation of the cellular differentiation [14,15]. It is triggered through 2 major signaling pathways [16-18]. The first involves death receptor family members, such as CD95/Fas, TRAILR1-2, TNF-R1, which in turn activate the caspases cascade, resulting in caspase 3 activation [19]. The process results in the proteolytic cleaveage of nuclear and cytoplasmic substrates, and the subsequent cellular disassembly [20,21]. Along with this Fas-dependent pathway, several stimuli, such as DNA damage, metabolic imbalance, growth factor deprivation, or cell cycle perturbation activates the alternative mitochondrial apoptotic pathway [18]. This implies that a very high number of signaling molecules involved in the processes may be altered causing an ALPS-like phenotype [22].

In conclusion, our study highlights the importance to evaluate Fas-induced cell survival in the clinical approach to patients with CAD even though the exact role of Fas-induced cell death abnormalities in the pathogenesis of CAD remains to be fully elucidated. The high prevalence of familiarity in such cases would suggest an inheritable pathogenetic mechanism, even though in previous studies it has been shown that there is no correspondence in the clinical phenotype among different family members indicating a role for several environmental and genetic factors [10,23].

## Competing interests

The authors declare that they have no competing interests.

## Authors’ contributions

LP participated in the study design, analysis and interpretation of data and wrote the draft of the manuscript. GG, FS have been involved in the collection of clinical data of the patients and in the interpretation of data. UR and UD carried out the molecular studies. CP designed and supervised the study. CP also wrote and approved the final version to be published. All authors read and approved the final manuscript.
